# A Comprehensive Availability Modeling and Analysis of a Virtualized Servers System Using Stochastic Reward Nets

**DOI:** 10.1155/2014/165316

**Published:** 2014-08-05

**Authors:** Tuan Anh Nguyen, Dong Seong Kim, Jong Sou Park

**Affiliations:** ^1^Department of Computer Engineering, Korea Aerospace University, 76 Hanggongdaehang-ro, Deogyang-gu, Goyang-si, Gyeonggi-do 412-791, Republic of Korea; ^2^Department of Computer Science and Software Engineering, College of Engineering, University of Canterbury, Private 4800, Christchurch 8140, New Zealand

## Abstract

It is important to assess availability of virtualized systems in IT business infrastructures. Previous work on availability modeling and analysis of the virtualized systems used a simplified configuration and assumption in which only one virtual machine (VM) runs on a virtual machine monitor (VMM) hosted on a physical server. In this paper, we show a comprehensive availability model using stochastic reward nets (SRN). The model takes into account (i) the detailed failures and recovery behaviors of multiple VMs, (ii) various other failure modes and corresponding recovery behaviors (e.g., hardware faults, failure and recovery due to Mandelbugs and aging-related bugs), and (iii) dependency between different subcomponents (e.g., between physical host failure and VMM, etc.) in a virtualized servers system. We also show numerical analysis on steady state availability, downtime in hours per year, transaction loss, and sensitivity analysis. This model provides a new finding on how to increase system availability by combining both software rejuvenations at VM and VMM in a wise manner.

## 1. Introduction

Computing systems with virtualization are rapidly gaining strong attention for computational sustainability by administrators of information resources in enterprises. Computational sustainability is a field to develop computational models, methods, and tools to help balance environmental, economic, and societal needs for a sustainable development [1]. Thus, virtualized computing systems, such as in software defined data center (SDDC) or infrastructure as a service (IaaS) in cloud computing, are core approach and promising solution to create a sustainable IT business infrastructure [1–3]. The IT business infrastructure with virtualization is capable to confront with variety of security concerns [4] as well as to avoid interruption of ordinary business processes [3, 5] and to assure high availability and continuity of information resources flowing within an organization [6]. In an IT business infrastructure, server virtualization is one of the essential parts of virtualization process along with storage virtualization, network virtualization, and workload management. Enterprises can save capital, floor space and energy via server virtualization and are able to improve business efficiencies due to resource utilization and autonomous management for heterogeneous workloads in data centers. The main idea behind server virtualization is to consolidate multiple workloads onto fewer physical servers (hereinafter, called host) with software based orchestration by creating multiple virtual servers (i.e., virtual machines (VM)) on a virtual machine monitor (VMM) in a physical host. In recent years, IT enterprises have also adopted server virtualization as the most appropriate approach in IaaS for cloud computing services to provide agile computing resources over the Internet. Cloud providers offer predescribed configuration of computing resources to cloud customer in accordance with service level based agreements (SLA) by assigning corresponding configuration of VM. Assuring high availability of cloud services over virtualization is of paramount importance. Thus, availability management and fault tolerance in such virtualized servers system are getting more concerned in both hardware and software aspects. High availability (HA) solutions [6–8] and fault tolerant mechanisms [9–11] have been proposed to counteract with hardware or software faults in virtualized servers system. Nevertheless, the studies individually do not take into account various failure modes in a complete manner. Also, a small number of works studied availability of virtualized servers systems in a quantitative way. Thus, it is necessary to incorporate various hardware and software failure modes along with corresponding recovery behaviors and analyze the availability of such systems.

The main drawbacks of previous work are that most of virtualized systems are composed of only one VM running on one VMM in a physical server; see the papers [9, 10]. This architecture is commonly used in modeling and analysis of a virtualized server system in a number of studies, even though the proposed architecture in their hypothesis shows multiple VMs hosted on one VMM [11, 12]. Some studies [10, 13, 14] did take into account an additional physical host under active/cold standby or active/passive configurations [6], but only for assessing the effectiveness of live migration of a VM. Moreover, the previous work has not properly captured the behaviors of a virtualized system with multiple VMs running on multiple physical host servers. Only a few papers considered thoroughly the involvement of both hardware and software failure modes.

The main contributions of this paper are summarized as follows.

- Studied a virtualized servers system with two VMs running on one VMM in each host, which is the active/active HA configuration [6].
- Incorporated various failure and recovery behaviors including hardware failure, software aging failure, and Mandelbug related failure.
- Captured different types of hardware and software dependencies: (i) between a physical host and hosted VMM, (ii) between VMM and VMs, and (iii) between VMs and a storage area network (SAN).
- Analyzed and found out (i) the use of a frequent rejuvenation on VM may lower steady state availability (SSA) of the virtualized systems whereas that of VMM rejuvenation may enhance the SSA; (ii) the frequent rejuvenation policy on VM is the main culprit of VM transaction loss; (iii) a proper combination of VM rejuvenation may enhance the SSA compared to that of VMM rejuvenation.

The rest of this paper is organized as follows. Related work is presented in Section 2.  Section 3 introduces a virtualized servers system.  Section 4 presents SRN models for the virtualized servers system. The numerical analysis and discussion are presented in Section 5. Finally, Section 6 concludes the paper.

## 2. Related Work

Server virtualization is now a mainstream technology offering a way to consolidate servers and enable autonomic management of heterogeneous workloads. Virtualized server systems may be composed of an overall architecture that is even more complex than that of traditional nonvirtualized server systems. According to virtualization concept, applications (hereafter, App) and operating system (OS) are encapsulated in a separate and completely isolated container called a virtual machine (VM), decoupled from the physical host by a hypervisor or virtual machine monitor (VMM) [13]. In virtualized server systems, a VM (i.e., virtual server) is a software implementation executing programs like a real server. Multiple VMs are designed to work simultaneously on one physical host regardless of different types of workloads. Therefore, instead of operating many servers at low utilization, virtualization squeezes more processing powers onto fewer servers running at higher level of total resource utilization. In previous literature [14], two types of server virtualization implementation have been presented: (i) hosted hypervisor running on a host operating system that provides virtualization services (e.g., Microsoft Virtual Server 2005  [15]); (ii) native or bare metal hypervisor running directly on system hardware (e.g., Microsoft Hyper-V [16], Citrix Xen [17], and VMWare ESX [18]). The native hypervisor implementation for server virtualization has been adopted in various studies on server systems  [19–22] since this approach facilitates faster transactions with hardware devices [13]. Thus, this implementation approach is also adopted in this paper.

Software rejuvenation was first introduced by Huang et al. [23] as a promising solution to mitigate the adverse effects of software aging. The main idea behind software rejuvenation is to gracefully terminate and periodically or adaptively restart the software execution environment in order to clear aging status. Hence, the technique aims to postpone or prevent the occurrence of aging-related failures under specific policies. Many different policies have been proposed to implement software rejuvenation on different systems. A profound classification of software rejuvenation techniques has been presented in detail by Alonso et al. [24]. Accordingly, software rejuvenation approaches can be classified in two main groups: time-based and inspection-based strategies. A software system with time-based rejuvenation policy is periodically rejuvenated every time as a predefined time interval has elapsed [25]. The rejuvenation process is triggered by a clock counting time [26, 27]. The determination of optimal interval to achieve maximum availability and minimum downtime cost, however, is mostly performed through building and analyzing an analytical model [27–29], whereas inspection-based rejuvenation is triggered in the case if aging effects measured through observations of system state violate restrict criteria or particular conditions. The rejuvenation trigger epoch is decided by a variety of mechanisms including threshold-based methods using aging indicators [30–32]; prediction-based approaches: machine learning, statistical approaches, or structural models  [33–36]; and mixed approaches using prediction methods to determine optimal threshold [37]. However, the implementation of inspection-based rejuvenation in a real environment could be troublesome for system administrator due to the growing complexity of the systems introduced by recent technologies (e.g., cloud computing) and heterogeneous environments (e.g., software defined data center) where the systems have to interact with each other. Previous literature showed that time-based rejuvenation associated with a proper scheduling technique could be a suitable solution for these scenarios. For instance, Naksinehaboon et al. [38] proposed efficient rejuvenation scheduling techniques for operating system/kernel rejuvenation combination between different nodes in a high computing system (HPC). Machida et al. [39] has presented a combined scheduling technique for server virtualization in a virtual data center.

Server rejuvenation was first used by Machida et al. in [39, 40] as a term to imply software rejuvenation implementation on a server. In nonvirtualized server systems, server rejuvenation is performed in a reboot of operating system to clear aging-related bugs. It is reported in [32, 41, 42] that aging phenomena do manifest in an operating system and cause performance loss, significant resource exhaustion, and unexpected system failures. The detection and analyses in the studies, however, are complicated and mostly employed in an evaluation process of operating system rather than during software execution. In virtualized server systems, server rejuvenation refers to a combined-rejuvenation scheduling technique to perform rejuvenation processes on both VMM and VM subsystems within a server or among servers under predetermined policies [39, 40]. There are a number of studies on rejuvenation strategies which are applied on virtualized server systems. Thein et al. [9, 29] modeled and analyzed a virtualized single-server system with multiple VMs. The study showed that the use of virtualization technology associated with software rejuvenation techniques can improve system availability in virtualized systems versus in nonvirtualized systems. However, the software rejuvenation in the study was implemented only on VM subsystem regardless of VMM subsystem involvement. The technique therefore can clear aging states of VMs and applications, except VMM. Since a VMM is hosting software, it is not rebooted frequently in a long-run period. Thus, the VMM subsystem suffers aging phenomena more easily than other parts of the system do, and the VMM performance degradation due to accumulation of aging-related bugs can influence more severely on the hosted VM's operation. Researchers have been still putting their efforts in finding a proper approach for software rejuvenation implementation on a virtualized server system in consideration of both VMM and VM subsystems. To resolve this issue, three VMM rejuvenation techniques have been proposed in consideration of hosted VMs' behaviors in works [10, 40, 43], namely, cold-VM rejuvenation, warm-VM rejuvenation, and migrate-VM rejuvenation. In the warm-VM rejuvenation, all hosted VMs are shut down prior to VMM rejuvenation regardless of the VMs' operational status. After VMM rejuvenation, the VMs are booted in sequence, whereas the implementation of warm-VM rejuvenation is based on the mechanisms of on-memory suspension and resume of VM's operating status, respectively, before and after VMM rejuvenation. The VMs' executions are suspended and stored in a shared memory system before triggering VMM rejuvenation. After the completion of VMM rejuvenation, the VMM reloads VMs' memory images in sequence to restore the VMs' executions. Instead of shutting down or suspending VMs as in the cold-VM or the warm-VM rejuvenations, the VM-migrate rejuvenation offers a VM live-migration approach in which all running VMs are migrated to another host prior to VMM rejuvenation and are migrated back to the former host as soon as the VMM rejuvenation completes. Machida et al. [10, 40] applied the above VMM rejuvenation techniques on VMM subsystem along with time-based rejuvenation on VM subsystem in a typical servers system consisting of one primary host (providing services) and another secondary host (for live migration of VMs). The primary host enables one VM to run on a VMM whereas the secondary host runs a VMM in awaiting state for the sake of the VM live migration. This host, however, is not taken into consideration in modeling and analysis. In this paper, we studies an extended architecture of a virtualized system in which the system consists of two virtualized hosts, each host has two VMs running on one VMM. And we attempt to model and analyze the system with the active involvement of both hosts in providing services. To avoid the complexity in modeling, we do not apply the known-above VMM rejuvenation strategies, which are not our main focus (we attempt to model and analyze the virtualized system in a complete manner regarding both hardware and software aspects). Instead, our approach is to clear all VMs' operating states during VMM rejuvenation. The clean VMs are booted in sequence after the completion of the VMM rejuvenation.

Two main analysis approaches including measurement-based approach and analytic modeling approach are usually applied to study virtualized server systems with time-based rejuvenation. The former approach collects empirical data of system operation and applies statistical analysis to determine the epoch over which to perform rejuvenation [41, 44], whereas the latter approach analyzes the system based on a set of analytical models such as partial model, system model, or hierarchical model [27, 40, 45, 46]. The models aim to capture failure modes and recovery behaviors by defining system states and transitions. However, various assumptions on failure and repair time distributions of state transitions need to be incorporated in the models as input parameters. The system characteristics are analyzed through a variety of output metrics, for instance, steady state availability, loss probability, or downtime cost. Also, in a virtualized system with software rejuvenation, the optimal rejuvenation schedule is determined by optimization techniques under particular criteria which are to maximize availability or to minimize downtime cost. In previous literature, some analytical techniques have been used to model and analyze a virtualized server system with software rejuvenation. Thein and Park [29] presented a recursive availability model using CTMC to capture the behavior of a virtualized system with a large number of VMs but the model did not incorporate VMM rejuvenation. In work [45], Kim et al. attempted to incorporate in a hierarchical stochastic model based on fault tree and CTMC the details of different hardware failures (CPU, memory, power, etc.), software failures (VMs, VMM, and application) and corresponding recovery behaviors. The study took into consideration the system architecture of two hosts with one VM running on one VMM in each host. But the modeling did not cover completely dependent behaviors between hardware and software subsystems due to the state explosion issue in CTMC modeling in the case of complex systems. Machida et al. [10, 40] presented comprehensive SRN availability models for VMM and VM in a server virtualized system with time-based rejuvenation. The models captured aging failure mode and applied time-based rejuvenation for both VMM and VM subsystems. Furthermore, the dependent behaviors between VMM and VM subsystems were taken into account in three cases of VMM rejuvenation techniques: cold-VM, warm-VM, and VM-migrate rejuvenations. In our work, we disregard VM live migration during VMM rejuvenation for simplicity. But we take into account in detail different hardware and software failure modes and recovery behaviors as well as dependent behaviors between subsystems. We attempt to analyze the impact of rejuvenation implementation on system availability of VM versus VMM subsystems in a typical virtualized system with multiple VMs.

## 3. A Virtualized Server System

### 3.1. System Architecture

**Figure 1:**
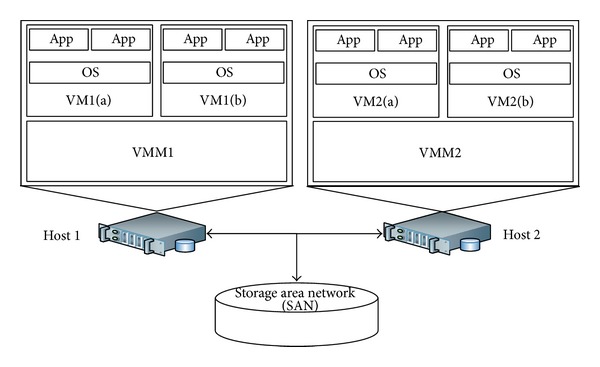
Architecture of a virtualized servers system.

The architecture of a typical virtualized servers system (VSS) with multiple VMs is depicted in Figure 1. The VSS consists of two physical servers (also called hosts, host1 and host2). Both hosts have an identical configuration. Each host has a VMM (which is also known as hypervisor) and each host runs two VMs on its VMM. Each VM subsystem is composed of an operating system (OS) and multiple identical applications (Apps) as wanted. In this paper, we disregard the involvement of OS, Apps, and workload, which has been studied in [47, 48]. The hosts share a storage area network (SAN) on which the VM images or VMM source code files are stored. We will be using this system to study availability of a virtualized system. The model can be further extended in the future, but our focus is to take into account the detailed behaviors of a virtualized system, in contrast to incorporating a large scale cloud system as in [49].

### 3.2. Failure Modes and Recovery Behaviors of the VSS

We take into account the following failure modes and corresponding recovery behaviors in SRN models to be presented in the next section.

- *Hardware failures* [45, 50] on hosts and SAN: both hosts are subject to hardware malfunctions due to hazardous faults on components (e.g., CPU, memory, disk, and cooler). Also, a SAN is likely exposed to hardware failures (e.g., failures of switches, disk array, tape, etc.). The hardware failures on hosts and SAN severely cause outage in operation of the subsystems. Once, the subsystems enter downtime state due to hardware failures, it is needed to summon a repairperson for hardware replacement or maintenance.
- *Nonaging-related Mandelbugs failures* [51] on both VMMs and VMs subsystems: Both VMM and VM subsystems apparently confront with software faults which are broadly divided into Bohrbugs and Mandelbugs [52]. A subtype of Mandelbugs, nonaging-related Mandelbugs (NAM), whose causes are unknown and can go unnoticed after the deployment of VMM and VM subsystems on a virtualized system. Therefore, the VMM and VM subsystems are likely incurred nonaging failures under the occurrence of NAM. In this scenario, a summoned repairperson has to investigate and fix the bugs thoroughly.
- *Software aging-related failures* [39, 53] on both VMMs and VMs subsystems: they are known as another subtype of Mandelbugs; software aging in long-run software systems like VMM and VM subsystems causes an increased failure rate and/or degraded performance due to accumulation of aging errors. The error condition brings a period of failure-probable state to bear on the VMM and VM subsystems in which the subsystems still run with degraded performance. If without external intervention, the subsystems inevitably undergo an aging-related failure [54]. Since then, a recovery process is conducted by a repairperson to remove aging causes and reconfigure the subsystems [55].
- But we do not incorporate* Bohrbugs* [56] in the VMMs and VMs subsystems, which are able to be found and removed in software development and testing phases.
- *Dependencies* are also taken into account in detail.
  - *Between host and VMM:*
    - if a host goes into failure state, in consequence, the running VMM (in robust or failure-probable states) falls into downstate in which the VMM subsystem no longer provides virtualization. The VMM in downstate is restarted to robust state as soon as the host is recovered to healthy state;
    - the VMM's operation, however, is suspended if the VMM currently resides in failure/rejuvenation states. After the failed host is repaired, a rollback and synchronization process (as adopting the active/active configuration [6]) is conducted to resume the VMM to the latest operational status which is logged and stored on SAN.
  - *Between VMM and VM:*
    - as the VMM enters either downstate or failure states, the hosted VM (in robust or failure-probable states) goes into downstate due to the consequence of its dependency on the hosting VMM. The VM in downstate is restarted to robust state when its VMM enters running states (either robust or failure-probable states);
    - if a VM is currently in failure/rejuvenation states, instead of pushing the VM to downstate as usual, a temporary VM suspension is performed. The current state of the VM including the state of all applications and processes running on the VM is saved into VM image file and stored on SAN. As soon as the hosting VMM enters running states (either robust or failure-probable states), the suspended VM is resumed by reloading the VM image file on SAN and it continues operating at its latest state;
    - furthermore, as the VMM rejuvenation process is triggered, the current states of all hosted VMs are cleared and reset to the clean state in which the VMs are ready to boot right after the completion of the VMM rejuvenation [43]. This strategy is to clean the whole virtualized system (including both VMM and VM subsystems) after every interval of VMM rejuvenation.
  - *Between VM and SAN:*
    - a VMM (as a hypervisor program) is loaded onto host's internal memory to execute without interruption and for higher performance [43]. However, VM image files (large size) are stored on SAN. Thus, the current operational state of SAN decides the running state of VM;
    - if the SAN fails, the VMs in running states (either robust or failure-probable states) go into downstate. A VM cannot restart unless the SAN is repaired;
    - if the current state of a VM is not in running states, we assume that its operation is suspended temporarily and resumed after the completion of SAN recovery.

### 3.3. Assumptions

In order to capture proper behaviors of VSS with multiple VMs, we made some assumptions as follows.

*(i) Distributions*. In order to make the analytical model as close as possible to a practical system, it is necessary to assume the distribution types of time to failure and time to recovery. However, there is no consensus on distributions in every failure mode and corresponding recovery behavior. Thus, it is better to apply general distributions but not restrict to predetermined ones for wide applicability. There is a large number of papers [10, 27–29, 39, 40, 57–60] in previous work supporting the use of exponential distribution. In this paper, we assume that the exponential distribution is generally applied on all transition times of timed transitions in the models. However, we assume to apply a deterministic distribution on time to trigger rejuvenations for both VMM and VM subsystems since the rejuvenation intervals are fixed values.

*(ii) Software Aging*. Through previous experiments, software aging has been reported as a phenomenon resulting into two cases: (i) sudden crash/hang failure [39, 61], which leads to software unavailability; (ii) progressive performance degradation [62–64]. However in this paper, both effects are considered in a single model and captured by the state of failure-probable in which the system manifests its degraded performance or high probability of failure.

*(iii) Unexpected Failure Events and Failover Mechanisms*. Since our focus is on detailed behavior of a virtualized system with multiple VMs and hosts, we restrict ourselves to not incorporate live VM migration and other failover mechanisms for the virtualized system which have been studied as in [27, 40, 47]. Also, to simplify the modeling, we do not consider any unexpected and unpredicted failure events during VMM/VM suspension and resume operations. These mechanisms and failure events in a virtualized system with multiple VMs are promising topics for future work.

*(iv) Monitoring Agents*. In most of system architectures in previous work [27, 36, 59, 65], several terms such as software rejuvenation agent (SRA), rejuvenation manager (RM), or management server were used in system architecture description as common components to monitor aging phenomenon and proceed to rejuvenation accordingly. It is supposed that our system does involve the above elements as a common management system to monitor and manage the operations of the virtualized system. However, since the above components are not taken into account in modeling as per previous studies, we therefore do not depict and describe the involvement of system management components for simplicity of system architecture presentation.

## 4. SRN Models of the VSS

### 4.1. SRN Model of a Multiple-VMs Virtualized Server System

**Figure 2:**
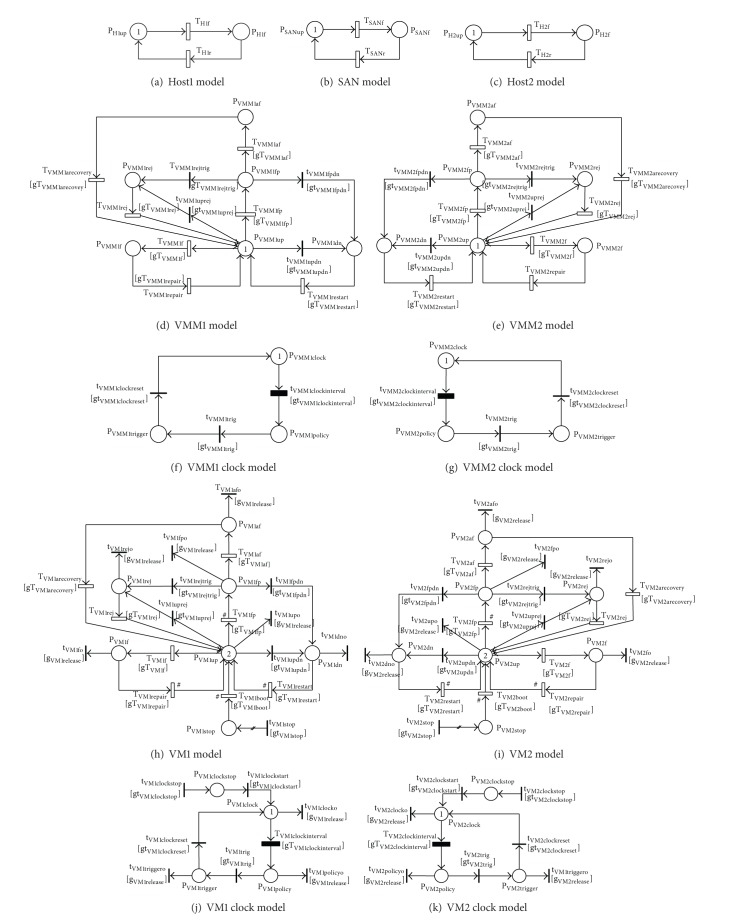
SRN models of a VSS with multiple virtual machines.

The entire SRN model of a VSS with multiple VMs is shown in Figure 2. The model is composed of partial SRN models of hosts, SAN, VMMs, and VMs derived from individual models in the next sections IV.B, IV.C, and IV.D. Figures 2(a)–2(k) depict, respectively, SRN models of host1, host2, SAN, VMM1, VMM2, VM1, and VM2. For the sake of time-based rejuvenation, each of VMM and VM models is correspondingly associated with a VMM clock or a VM clock. To actively control system behaviors and dependencies, a set of guard functions is attached to transitions in order to enable or disable the transitions under predetermined conditions. All guard function definitions in the system model can be consistently referred to the guard function definitions in the partial models (defined in Tables 1 and 2) with regard to the alteration of notations for the correspondingly attached transition and model. For example, we consider the guard function gT_VMMrestart_ attached to the transition T_VMMrestart_ in the VMM partial model. The notation of the above guard function in the VMM1 model (Figure 2(d)) is altered to gT_VMM1restart_. This function is attached to T_VMM1restart_, and its function definition is also altered accordingly. The above described alteration is applied consistently for all other guard functions, their definition, and notations in the system model.

### 4.2. Hosts and SAN Submodels

**Figure 3:**
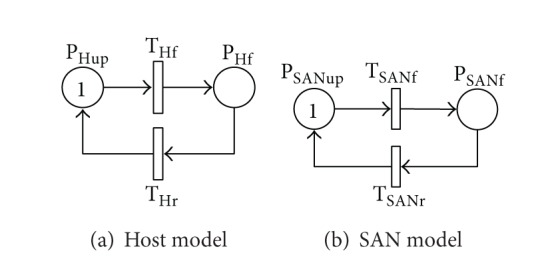
SRN models for host and SAN.

The failure and recovery behaviors of a host are represented as two places; up and failure in Figure 3(a). A host is in upstate represented by one token in P_Hup_. Because of hardware malfunctions, failure transition T_Hf_ is fired; the token in P_Hup_ is taken and deposited in P_Hf_; the host enters failure state. A failed host is repaired by summoning a repairperson and returns to upstate (P_Hup_). The repair transition T_Hr_ is enabled; the token in P_Hf_ is taken and deposited in P_Hup_.

Similarly, the failure and recovery behaviors of SAN are modeled as in Figure 3(b). The SAN is initially considered in upstate. As time goes by, the SAN fails due to hardware malfunctions, and its state becomes failure state (P_SANf_). After recovery by summoning a repairperson, the SAN returns to upstate (P_SANup_). When the SAN fails, T_SANf_ is fired; the token in P_SANup_ is taken and deposited in P_SANf_. As the SAN is repaired (T_SANr_ is enabled), the token in sequence is taken from P_SANf_ and deposited in P_SANup_.

### 4.3. VMM Models with Time-Based Rejuvenation

**Figure 4:**
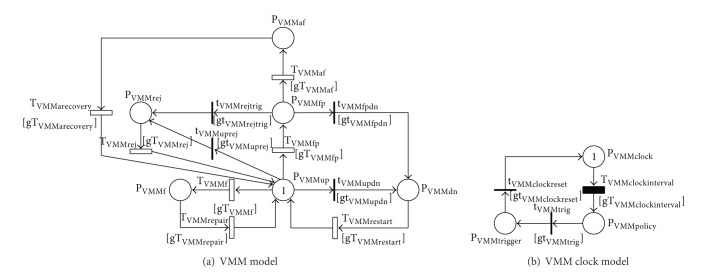
SRN models for a VMM subsystem.

A VMM subsystem with time-based rejuvenation is modeled as shown in Figure 4. The model consists of two submodels: (a) VMM model and (b) VMM clock model. The VMM model (Figure 4(a)) captures different failure modes and recovery actions including aging-related failure and time-based rejuvenation policy, failures due to nonaging-related Mandelbugs (NAM) and repair, and dependency of the VMM on its underlying host. The VMM clock (Figure 4(b)) is used to trigger time-based rejuvenation. The VMM is initially in up and running state (depicted by one token in P_VMMup_), in which the system is highly robust and works without errors. When a nonaging-related Mandelbug has appeared, the VMM goes into failure state (P_VMMf_). The failure transition T_VMMf_ is fired; the token in P_VMMup_ is taken and deposited in P_VMMf_. The repair is conducted by enabling T_VMMrepair_, and then the token is taken from P_VMMf_ and deposited in P_VMMup_. The repaired VMM returns to stable state (P_VMMup_). Besides, as time goes on, the VMM in upstate undergoes the aging period [39]. This phenomenon is captured by transiting through T_VMMfp_ one token from P_VMMup_ to P_VMMfp_. The VMM becomes failure-probable (the token in P_VMMup_ is taken and deposited in P_VMMfp_). If the VMM rejuvenation process is not triggered, the VMM goes through an aging-related failure from failure-probable state. The token in P_VMMfp_ is taken and deposited in P_VMMaf_. The recovery is captured by firing T_VMMarecovery_. The token in P_VMMaf_ is taken out and deposited in P_VMMup_. The VMM returns to the stable state P_VMMup_. In the case that the point of time for rejuvenation has approached, regardless of the VMM status (either in the failure-probable state (P_VMMfp_) or in the stable state (P_VMMup_)), time-based rejuvenation process of the VMM is triggered. This behavior is controlled by two guard functions gt_VMMrejtrig_ and gt_VMMuprej_. The immediate transitions t_VMMrejtrig_ and t_VMMuprej_ are enabled. The token in P_VMMup_ or P_VMMfp_ is taken and deposited in P_VMMrej_. The VMM enters rejuvenation-ready state (P_VMMrej_). Hereafter, the VMM is reset and undergoes a rejuvenation process. When this process completes, the transition T_VMMrej_ is enabled. The token in P_VMMrej_ is taken and deposited in the stable state (P_VMMup_). We also take into account the dependency of the VMM on its underlying host. If the host enters failure state, the VMM in stable state (P_VMMup_) or failure-probable state (P_VMMfp_) goes instantly to downstate (P_VMMdn_) through, respectively, either fired immediate transitions t_VMMupdn_ or t_VMMfpdn_. The token in P_VMMup_ or P_VMMfp_ is taken out and deposited in P_VMMdn_. This token transition is controlled by the guard functions gt_VMMupdn_ and gt_VMMfpdn_. As soon as the host returns to upstate, the VMM restarts via enabling T_VMMrestart_. The token in P_VMMdn_ is taken out and deposited in P_VMMup_. However, if the VMM is in failure states (P_VMMaf_ and P_VMMf_) or in rejuvenation-ready state (P_VMMrej_) as the host enters downstate, the repair/maintenance operations of the VMM suspend temporarily. The operational status of the VMM stored on the shared storage system is fetched to roll back to the former state as soon as the host returns to upstate.

In order to carry out time-based rejuvenation, we use the VMM clock. To count the time progressing and to ensure precise intervals for rejuvenation, we employ a deterministic transition, T_VMMclockinterval_, which takes the duration of 1/*τ*_VMM_ to fire. In order to implement the models on software package SPNP [66], we use c_VMM_-stage Erlang distribution to approximate the deterministic transition T_VMMclockinterval_. The condition for counting time is that the VMM is in operation, or in other words, one token exists either in P_VMMup_ or P_VMMfp_. At a specific interval, T_VMMclockinterval_ is enabled; the token in P_VMMclock_ is taken and deposited in P_VMMpolicy_. At this moment, the VMM clock triggers the VMM rejuvenation process as long as t_VMMtrig_ is enabled. Soon after the VMM enters rejuvenation-ready state P_VMMrej_, the VMM clock is reset to counting state and starts a new routine. Thus, t_VMMclockreset_ is enabled and the token in P_VMMtrigger_ is taken and deposited in P_VMMclock_.

The above dynamic behaviors of the VMM subsystem are controlled by a set of guard functions associated with respective transitions as listed in Table 1.

### 4.4. VM Models with Time-Based Rejuvenation

**Figure 5:**
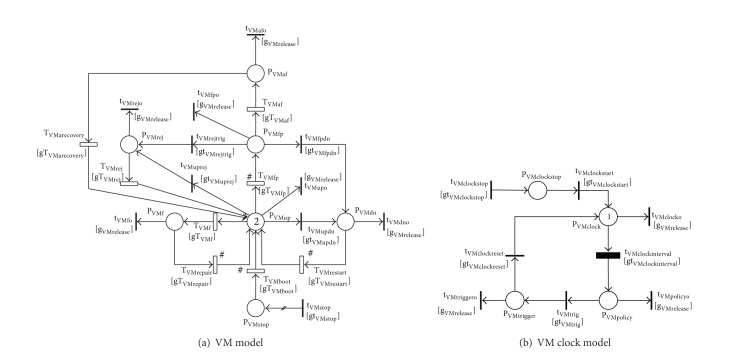
SRN models of VM subsystem.

SRN models for VM and VM clock are shown in Figures 5(a) and 5(b), respectively. Initially, each VMM has two running VMs in robust state, which are represented by two tokens in P_VMup_. The failure and recovery behaviors including the aging period, aging-related failure and recovery, nonaging-related Mandelbugs failure, and repair action are captured and described similarly to those in the VMM model (Figure 4(a)). We here describe the distinction of VM model. The dependency of VMs on VMM is captured in various cases of VMM failure modes. Moreover, the marking dependence between VMs and the dependence of VMs on SAN are also taken into account in this VM model.

The dependency between the running VM and its underlying VMM is captured in this model as follows. As long as the underlying VMM exists either in stable state (P_VMup_) or in failure-probable state (P_VMMfp_), the hosted VM can run uninterruptedly. If the VMM enters failure state or downstate, the hosted VM instantly goes to downstate (P_VMdn_) regardless of its operational states (P_VMup_ or P_VMfp_). The immediate transitions t_VMupdn_ and t_VMfpdn_ fire and the token either in P_VMup_ or in P_VMfp_ is taken out and deposited in P_VMdn_. The failed VM can only restart after the underlying VMM returns to running states (P_VMup_, P_VMMfp_). However, if the VM is in failure states (P_VMf_, P_VMaf_) or rejuvenation-ready state (P_VMrej_) as the VMM enters failure states or down state, the VM's operations are suspended. Its operational status is stored on shared storage system. After the VMM returns to running states, the former operational state of the VM is rolled back. We also incorporate the dependency between the VMM and the hosted VMs during VMM rejuvenation. When the VMM is under rejuvenation, the current states of VM and VM clock are cleaned and reconfigured to be ready to boot/start after the completion of the VMM rejuvenation. A set of immediate transitions (t_VMupo_, t_VMfpo_, t_VMdno_, t_VMafo_, t_VMrejo_, t_VMfo_) is fired to clear the current states of the VM system by removing all tokens in respective input places in VM model (see Figure 5(a)). Also, the immediate transitions t_VMclocko_, t_VMpolicyo_, t_VMtriggero_ are used to remove tokens in their respective input places in VM clock model in order to clear the current states of the VM clock model (see Figure 5(b)). The VM clock is stopped by firing the transition t_VMclockstop_ and depositing only one token in P_VMclockstop_. To ensure that the two VMs are stopped and cleaned to their initial state, only two tokens at most can be deposited in P_VMstop_ through t_VMstop_. Therefore, an input multiplicity arc is used to flexibly adjust the number of tokens deposited in P_VMstop_ upon the current number of tokens existing there. If there is no token in P_VMstop_, the arc allows two tokens at most to be deposited in P_VMstop_. If the number of tokens existing in P_VMstop_ is one, the arc enables to deposit only one token in P_VMstop_. To implement this, a cardinality arc function *marc*_VMstop_ is designed to control the number of tokens deposited in P_VMstop_ through the multiplicity arc. When the underlying VMM returns to stable state (P_VMMup_) after rejuvenation and exists in running states (P_VMMup_ and P_VMMfp_), it restarts each VM in sequence by enabling the transition T_VMboot_. The tokens in P_VMstop_ are taken out one by one and deposited in P_VMup_. A long with the completion of booting a VM, the VM clock also starts counting time as soon as t_VMclockstart_ is fired and the token in P_VMclockstop_ is taken and deposited in P_VMclock_. Furthermore, there are some dependent cases in which two VMs all exist in the same state such as P_VMdn_, P_VMf_, P_VMstop_, or P_VMup_ which, respectively, need to restart (T_VMrestart_), to repair (T_VMrepair_), to boot (T_VMboot_), or are going to be failure-probable (T_VMfp_). In these cases, all VMs compete to each other to enter a new state. For this reason, a dependency between VMs called marking dependence is necessary to be incorporated in the modeling since this dependency affects the rate of the transitions. A sign “#” is placed next to every output transition of the mentioned places to imply that a marking dependence is associated to related transitions (see Figure 5). The time to trigger VM rejuvenations is captured by using a deterministic transition, T_VMclockinterval_, in VM clock model. The deterministic transition is fired after every interval of 1/*τ*_VM_. We use c_VM_-stage Erlang distribution for the deterministic transition T_VMclockinterval_. The definition of guard functions is depicted as in Table 2.

## 5. Numerical Results and Discussions

We implemented the SRN models in stochastic Petri net package (SPNP) [66]. In order to study system characteristics in terms of business availability and continuity featured for computational sustainability in an IT business infrastructure, we analyzed the following metrics: steady-state availability (SSA), transaction loss, and sensitivity of the SSA with respect to clocks' interval.  Table 3 summarizes the parameter default values, based on previous works [10, 45].

### 5.1. Steady-State Availability Analysis

We first computed the SSA of the VSS using the default parameters' value. We conducted numerical experiments in seven case studies with regard to different rejuvenation combinations. The case studies are described along with notations in Table 4. The results are summarized as in Table 5.

The SSAs are abnormally not the highest as expected even though a combined-rejuvenation countermeasure is adopted simultaneously on both VMM and VM subsystems. This is because of the improper strategy of rejuvenation operations between VMM and VM subsystems. Furthermore, the presence of VMM rejuvenation has positive impact on system availability versus negative impact of the presence of VM rejuvenation strategy. This means, the presence of rejuvenation on VMM subsystems enables the system to gain SSA but inversely for the presence of rejuvenation on VM subsystems. This is derived by comparing SSAs in the cases with/without rejuvenation on VMM/VM subsystems. This phenomenon can be explained as the consequence of a frequent rejuvenation policy on VM subsystems in a system with multiple VMs. Also, it is because of inflexible and uncoordinated rejuvenation policies between both VMM and VM levels causing the side effect. Although this study reflects the abnormal role of rejuvenation policies on VMMs and VMs under given parameters in Table 3, we still recommend adopting rejuvenations at both VMM and VM levels thoroughly to avoid long-run system malfunctions because of software aging. The coordination of rejuvenation policies on each individual of VMMs and VMs requires more in-depth studies.

### 5.2. Transaction Loss

We use the following metrics to evaluate VMs subsystem downtime: total downtime in hours per year and mean time to failure equivalent (MTTFeq) as shown in Table 6. The total number of hours in a year of VMs subsystem downtime is about 72 hours, whereas the meantime between each failure of VMs subsystem is approximately at 218 hours. Furthermore, we took into consideration some main causes of transaction losses to compute expected number of transaction losses per year of VMs subsystem as in Table 7. We evaluate VMs transaction loss in three cases: (i) VSS with both VMM and VM rejuvenation; (ii) VSS without VM rejuvenation but with VMM rejuvenation; and (iii) VSS without VMM rejuvenation but with VM rejuvenation. Our analysis discussion is conducted as in the following major points.

- Under the default value of input parameters, the main culprit of VMs transaction losses is VM rejuvenation. The VM rejuvenation contributes the most of transaction losses which are relatively at 83.28% and 93.53% of total number of VM transaction losses, respectively, in the cases (i) and (iii) which are with and without VMM rejuvenation. The reason of the above side effect is that the frequent VM rejuvenation actions drastically reset the four VMs in either robust or aging states periodically at predetermined intervals regardless of operational efficiency and coordination. This is to imply the negative implications of improper VM rejuvenation actions in a virtualized system with multiple VMs.
- However, if without VM rejuvenation, the aging-related failure on VMs subsystem occurs much more often. This is shown as follows. The ratio of transaction losses due to VM aging failure increases from about 2.05% up to 27.66% of total number of VM transaction losses, respectively, in the cases of with and without VM rejuvenation (cases (i) and (ii)). Accordingly, the number of VM transaction losses per year increases almost three times from about 33.8 up to 92.2 in respective cases, while the number and the ratio of transaction losses due to VM aging failure change slightly in the cases (i) and (iii) which are, respectively, with and without VMM rejuvenation. This again points out the negative impact of improper VM rejuvenation when the virtualized system hosts multiple VMs.
- Apparently shown in Table 7, if without VMM rejuvenation (case (iii)), the number of VM transaction losses per year increases from about 38.9 in the case (i) (with VMM rejuvenation) up to 56.4 in the case (iii) (without VMM rejuvenation). This is clearly due to VMM aging failure. Without VMM rejuvenation, the VMMs likely undergo VMM aging-related failure, which extend the VMM downtime. Therefore, the number of VM transaction losses also increases as VMM rejuvenation is not applied. However, the presence of VMM rejuvenation also contributes a portion of VM transaction losses which is about 197 per year. The reason is due to the method used to deal with the hosted VMs during VMM rejuvenation. As VMM rejuvenation proceeds, the process not only rejuvenates VMM subsystem but also cleans VMs subsystem regardless of its current operational states. Without failover mechanisms, this policy causes VM transaction losses although VMs are in running states (robust or failure-probable states).

### 5.3. Sensitivity Analysis

**Figure 6:**
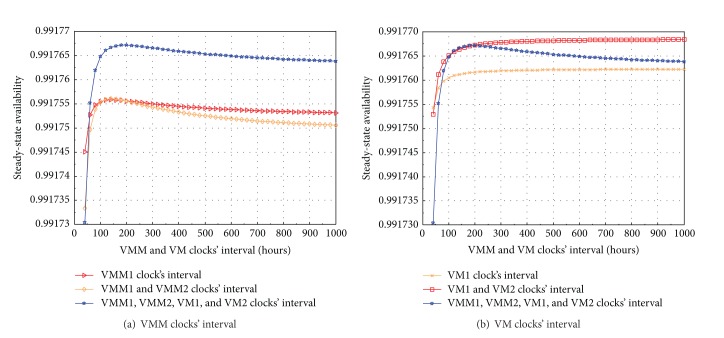
SSA sensitivity analysis of VM subsystem with respect to VMM and VM clocks' interval.

The above SSA analysis and transaction loss analysis reveal complicated behaviors and characteristics of a virtualized system with multiple VMs. Hereby there is a critical need to analyze and seek for a proper combination of VMM and VM rejuvenations. In order to study particular affections of each combination of rejuvenations, we perform sensitivity analysis of system's SSA.  Figure 6 shows the results of SSA analysis by varying rejuvenation clocks' interval of VMM and VM subsystems. The sensitivity analysis is observed in 5 case studies with respect to the variation of (i) only VMM1 clock's interval; (ii) only VM1 clock's interval; (iii) both VMM1 and VMM2 clocks' interval; (iv) both VM1 and VM2 clocks' interval; and (v) all clocks' interval with the same duration. The interval values range in 0–1000 hours for experiment while other parameter values are fixed. It is apparent in the analysis results that there is a common variation tendency for all case studies. In the early period (0–200 hours), if we assign an increased value of clocks' interval, the SSA of system significantly increases. But after that, the more the value of clocks' interval increases, the more the SSA appears to drop.  Figure 6(a) shows the SSA sensitivity with respect to the variation of VMM clocks' interval. It is very interesting that the rejuvenations on both VMM subsystems in two hosts (rhombus shaped line) with the same interval values are not an ideal solution compared to the rejuvenation only on one of the two VMMs (triangle shaped line). However, if the rejuvenations are conducted on both VMM subsystems and also together on both VM subsystems (star shaped line), the SSA is enhanced clearly. This pinpoints the role of rejuvenations with long intervals on VM subsystems in a system with multiple VMs.  Figure 6(b) shows the SSA sensitivity with respect to the variation of VM clocks' interval. In this case, the rejuvenations on both VM subsystems (rectangle shaped line) enable the system to gain clearly higher SSA compared to the rejuvenation only on one of VM subsystems (cross shaped line) and even relatively higher compared to the rejuvenations on all VMMs, VMs subsystems (star shaped line). But it is not much different in early period of rejuvenation interval range (0–200 hours) in the comparison between the case of rejuvenations on both VMs and the case of rejuvenations on all VMMs and VMs.

**Figure 7:**
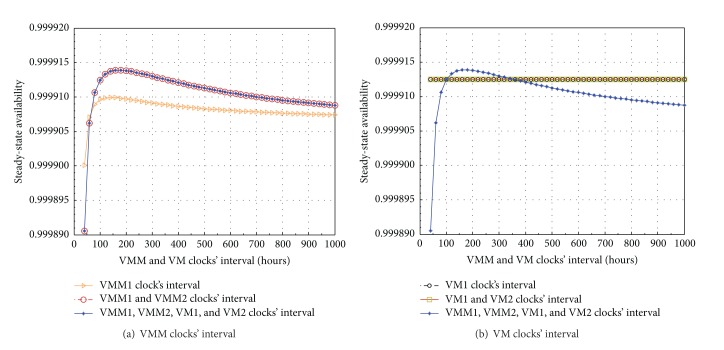
SSA sensitivity analysis of VMM subsystem with respect to VMM and VM clocks' interval.

We extend our sensitivity analysis of the SSA for VMM subsystem with respect to VMM and VM clocks' interval as showed in Figure 7. The sensitivity analysis is also performed in 5 case studies with the same settings as in the sensitivity analysis for VM subsystems. Comparing both sensitivity analyses showed in Figures 6 and 7, we find that the variation tendencies of the SSA in both analyses are similar to each other; however, the SSA values of VMM subsystem are always much higher compared to those of VM subsystem (the SSAs vary in the range of [0.999890–0.99915] for VMM subsystems and [0.991730–0.991770] for VM subsystems). Furthermore, the SSA sensitivity analysis of VMM subsystems in Figure 7 apparently reflects the dependency between VM subsystems and VMM subsystems in which the variations of VM clocks' interval do not affect the SSA of VMM subsystems. In Figure 7(a), the variations of VMM and VM clocks' interval in two cases, (iii) VMM1 and VMM2 clocks' interval (circle shaped line) and (v) all clocks' interval (star shaped line), bring about the same SSA analysis results of VMM subsystem (two lines overlap to each other). This points out that the involvement of the variation of VM clocks' interval does not affect the SSA of VMM subsystem. This phenomenon is reflected more clearly in Figure 7(b) in which the variations of VM clocks' interval in two cases, (ii) VM1 clock's interval (black circle shaped line) and (iv) VM1 and VM2 clocks' interval (rectangle shaped line), do not even change the SSA values of VMM subsystem (both lines horizontally overlap). Whereas in Figure 6, the variations of VMM clocks' interval do affect and the variations of VM clocks' interval strongly affect the SSA of VM subsystems. This argument reflects that the VM subsystems do depend on the VMM subsystems but the VMM subsystems do not depend on the VM subsystems. Nevertheless, the dependency of the VMM subsystems on the VM subsystems could be a fruitful topic for future extension. In Figure 7(a), we also find that the variations of both VMM clocks' interval in the case (iii), VMM1 and VMM2 clocks' interval, do enhance the SSA of VMM subsystems compared to those of only one VMM clock's interval in the case (i): VMM1 clock's interval.

Based on the above SSA sensitivity analyses for both VMM and VM subsystems with respect to corresponding VMM and VM clocks' interval, we recommend that system administrators should rejuvenate all VMM and VM subsystems with the value of intervals in the range [150–200] hours to gain high SSA.

### 5.4. Limitation and Discussions

There are a number of research issues remaining open to improve as follows.

- In our system, the VMs' operational states are cleared and reset to clean state during VMM rejuvenation regardless of VMs' current status. This policy, however, drastically pushes a VM in running states (either robust state or failure-probable state) into downstate. Therefore, it could cause more VM transaction losses. Thus, a proper failover mechanism such as live VM migration can be considered as a mandatory measure in the virtualized system with multiple VMs to enhance significantly system availability. This idea still remains open for further extension of our work.
- In our work, we neglected unexpected failure events during VMM/VM suspension or resume operations. But in reality, these operations could face a number of failure events regarding hardware and software aspects. Thus, there is still a need to light up this shadow corner in the empirical or analytical studies of virtualized system with multiple VMs.
- In order to investigate detailed behaviors of time-based rejuvenation process on the virtualized system, in our modeling, we attempted to separate two VMMs and attach a VMM clock to trigger VMM rejuvenation process onto each VMM. But we did not separate two VMs on each VMM yet. Thus, the two VMs use the same VM clock to trigger VM rejuvenation process. However, in reality each VM could be equipped with its own clock so that each VM could be monitored individually and rejuvenated separately in flexible rejuvenation strategies. This approach, nevertheless, need to be considered carefully regarding the types of stochastic model to avoid complicated and explosive modeling.

### 5.5. Future Research Avenue

Beyond the limitations and improvement opportunities in subsection D, we find a fruitful future research avenue for our work.

- Our work has done the sensitivity analysis of the SSA of both VMM and VM subsystems with respect to VMM and VM clocks' interval. Nevertheless, it is clear that a comprehensive sensitivity analysis can be performed with respect to many other parameters of the system. Thus, there is an open way to observe the VSS behaviors based on a set of parameters in order to gain higher interests.
- In our work, we divide a very large and expected-to-build monolithic model into several submodels of every entity in the VSS system. We use SRN to construct individual submodels. By manipulating a set of guard functions attached to transitions, we make the SRN submodels interact to each other to capture the dependencies and complex behaviors within the whole system. Our focus is to develop a very detailed and comprehensive availability model rather than constructing a very large scale monolithic availability model. Thus, we attempt to observe the VSS as a unit in complex, actual systems with a large number of VSS nodes. From this point, we find an open future research avenue to scale up the complexity of the current system to a complex, actual systems composed of tens, hundreds of nodes. However, it is common to confront with the state-space explosion problem using Markovian models like the SRN as well as the difficulties of system model integration in large scale virtualized systems. To reduce the complexity of such large scale systems, we may follow the same approach in this paper. We can divide the overall model into submodels; with iteration over individual submodels we can obtain the overall solution for the whole system. Also, proper interactions between submodels need to be taken into consideration into the iterative overall solution. For further details, see [49, 67] for the works on the scalable availability SRN models and interacting Markov chain models of the real case study of infrastructure-as-a-service cloud (IaaS). This paper could be extended with similar approaches for future work.
- The model in this study is based on the exponential distribution and Erlang distribution attached to transitions. However, in an actual virtualized system especially a system composed of both hardware and software components being modeled, many system behaviors do not conform to exponential distribution but nonexponential distribution, like hardware and software aging phenomena. Furthermore, the SRN model of the VSS in this paper is automatically converted to Markov reward model to be solved. A realistic virtualized system with many complex behaviors such as time-dependent rates, nonexponential distributions, and aging effects, however, cannot be modeled and captured by Markovian models but by non-Markovian models using discrete state-space methods. The methods allow to model and analytically evaluate any kind of dependability static and dynamic behaviors. Therefore, further work on incorporating nonexponential distribution and applying non-Markovian models for virtualized servers systems is an important endeavor. For more detail on nonexponential distribution, discrete state-space methods, and non-Markovian models in system dependability evaluation, see [68].

## 6. Conclusions

We have modeled and analyzed a virtualized servers system with multiple VMs via SRN. We encapsulated four VMs running on two VMMs into two hosts. We also incorporated diverse failure modes and corresponding recovery behaviors regarding hardware and software aspects including host failure, SAN failure, aging-related failure, and Mandelbugs related failure in SRN models. A variety of dependencies were taken into account in modeling as follows: (i) dependencies between a host and its hosted VMM, in turn between the VMM and its hosted VMs; (ii) interconnection dependency between SAN and VM subsystems; and (iii) marking dependency between VMs in a host. The SSA analysis showed that a frequent rejuvenation policy on VM may lower the SSA of the virtualized systems whereas that on VMM may enhance the system SSA. Based on the sensitivity analysis with respect to SSA, we showed that adopting a particular combination of rejuvenations on all VMM and VM subsystems in both hosts with the value of common interval in a specific range may help to increase system availability of the virtualized system.

## Figures and Tables

**Table 1:** Guard functions for the VMM submodel and VMM clock submodel.

Guard function name	Associated transition	Definition
gT_VMMrestart_	T_VMMrestart_	If (#P_Hup_ == 1) 1 else 0
gt_VMMupdn_	t_VMMupdn_	If (#P_Hf_ == 1) 1 else 0
gT_VMMf_	T_VMMf_	If (#P_Hup_ == 1) 1 else 0
gT_VMMrepair_	T_VMMrepair_	If (#P_Hup_ == 1) 1 else 0
gT_VMMfp_	T_VMMfp_	If (#P_Hup_ == 1) 1 else 0
gT_VMMaf_	T_VMMaf_	If (#P_Hup_ == 1) 1 else 0
gT_VMMarecovery_	T_VMMarecovery_	If (#P_Hup_ == 1) 1 else 0
gT_VMMfpd_	T_VMMfpd_	If (#P_Hf_ == 1) 1 else 0
gT_VMMinterval_	T_VMMinterval_	If (#P_VMMup_ == 1 || #P_VMMfp_ == 1) 1 else 0
gt_VMMtrig_	t_VMMtrig_	If (#P_VMMup_ == 1 || #P_VMMfp_ == 1) 1 else 0
gT_VMMclockinterval_	T_VMMclockinterval_	If (#P_VMMup_ == 1|| #P_VMMfp_ == 1) 1 else 0
gt_VMMclockback_	t_VMMclockback_	If (#P_VMMaf_ == 1 || #P_VMMf_ == 1 || #P_VMMdn_ == 1) 1 else 0
gT_VMMrejtrig_	T_VMMrejtrig_	If (#P_VMMtrigger_ == 1) 1 else 0
gt_VMMuprej_	t_VMMuprej_	If (#P_VMMtrigger_ == 1) 1 else 0
gt_VMMclockreset_	t_VMMclockreset_	If (#P_VMMrej_ == 1) 1 else 0
gT_VMMrej_	T_VMMrej_	If (#P_Hup_ == 1) 1 else 0

**Table 2:** Guard functions for VM model and VM clock model.

Guard function	Transition	Definition
gT_VMf_	T_VMf_	If (#P_VMMup_ == 1 || #P_VMMfp_ == 1 && #P_SANup_ == 1) 1 else 0
gT_VMrepair_	T_VMrepair_	If (#P_VMMup_ == 1 || #P_VMMfp_ == 1 && #P_SANup_ == 1) 1 else 0
gt_VMupd_	t_VMupd_	If (#P_VMMdn_ == 1 || #P_VMMf_ == 1 || #P_VMMaf_ == 1 || #P_VMMrej_ == 1 || #P_SANf_ == 1) 1 else 0
gT_VMrestart_	T_VMrestart_	If (#P_VMMup_ == 1 || #P_VMMfp_ == 1 && #P_SANup_ == 1) 1 else 0
gT_VMfp_	T_VMfp_	If (#P_VMMup_ == 1 || #P_VMMfp_ == 1 && #P_SANup_ == 1) 1 else 0
gT_VMaf_	T_VMaf_	If (#P_VMMup_ == 1 || #P_VMMfp_ == 1 && #P_SANup_ == 1) 1 else 0
gT_VMarecovery_	T_VMarecovery_	If (#P_VMMup_ == 1 || #P_VMMfp_ == 1 && #P_SANup_ == 1) 1 else 0
gT_VMfpdn_	T_VMfpdn_	If (#P_VMMdn_ == 1 || #P_VMMf_ == 1 || #P_VMMaf_ == 1 || #P_VMMrej_ == 1 || #P_SANf_ == 1) 1 else 0
gT_VMinterval_	T_VMinterval_	If (#P_VMup_ == 1 || #P_VMup_ == n_VM_ || #P_VMfp_ == 1 || #P_VMfp_ == n_VM_) 1 else 0
gt_VMclockback_	t_VMclockback_	If (#P_VMup_ == 0 && #P_VMfp_ == 0) 1 else 0
gt_VMtrigger_	t_VMtrigger_	If (#P_VMup_ == 1 || #P_VMup_ == n_VM_ || #P_VMfp_ == 1 || #P_VMfp_ == n_VM_) 1 else 0
gt_VMclockreset_	t_VMclockreset_	If (#P_VMrej_ == 1 || #P_VMrej_ == n_VM_) 1 else 0
gT_VMfprejtrig_	T_VMfprejtrig_	If (#P_VMtrigger_ == 1) 1 else 0
gt_VMuprej_	t_VMuprej_	If (#P_VMtrigger_ == 1) 1 else 0
gT_VMrej_	T_VMrej_	If (#P_VMMup_ == 1 || #P_VMMfp_ == 1 && #P_SANup_ == 1) 1 else 0
gT_VMboot_	T_VMboot_	If (#P_VMMrej_ == 0 && #P_VMMup_ == 1 || #P_VMMfp_ == 1) 1 else 0
gt_VMstop_	t_VMstop_	If (#P_VMMrej_ == 1 && #P_VMstop_ < n_VM_) 1 else 0
g_VMrelease_	t_VMupo_, t_VMfpo_, t_VMafo_, t_VMrejo_, t_VMsdno_, t_VMfo_, t_VMclocko_, t_VMpolicyo_, t_VMtriggero_	If (#P_VMMrej_ == 1) 1 else 0
gt_VMclockstart_	t_VMclockstart_	If (#P_VMMrej_ == 0 && #P_VMMup_ == 1 || #P_VMMfp_ == 1 && #P_VMclock_ == 0 && #P_VMclockstop_ == 1) 1 else 0
gt_VMclockstop_	t_VMclockstop_	If (#P_VMMrej_ == 1 && #P_VMclockstop_! = 1) 1 else 0
*ma* *rc*_VMstop_	t_VMstop_	If (#P_VMstop_ < n_VM_) (n_VM_-#P_VM1stop_) else 0
gT_VMclockinterval_	T_VMclockinterval_	If (#P_VMup_ == 1 || #P_VMup_ == n_VM_ || #P_VMfp_ == 1 || #P_VMfp_ == n_VM_) 1 else 0
gT_VMMfpdn_	T_VMMfpdn_	If (#P_Hf_ == 1) 1 else 0
gt_VMupdn_	t_VMupdn_	If (#P_VMMdn_ == 1 || #P_VMMf_ == 1 || #P_VMMaf_ == 1 || #P_VMMrej_ == 1 || #P_SANf_ == 1) 1 else 0
gT_VMd_	T_VMd_	If (#P_VMpolicy_! = c_VM_ && #P_SANup_ == 1 && #P_VMMup_ == 1 || #P_VMMfp_ == 1) 1 else 0
gT_VMdrej_	T_VMdrej_	If (#P_VMpolicy_! = c_VM_ && #P_SANup_ == 1 && #P_VMMup_ == 1 || #P_VMMfp_ == 1) 1 else 0
gT_VMd_	T_VMd_	If (#P_VMpolicy_! = c_VM_ && #P_SANup_ == 1 && #P_VMMup_ == 1 || #P_VMMfp_ == 1) 1 else 0
gT_VMdrej_	T_VMdrej_	If (#P_VMpolicy_ == c_VM_ && #P_SANup_ == 1 && #P_VMMup_ == 1 || #P_VMMfp_ == 1) 1 else 0

**Table 3:** Input parameter values used in the analysis.

Parameters	Description	Assigned transitions	Mean time/values
*μ*_hr_	Host repair rate	T_H1r_, T_H2r_	3 days
*λ*_hf_	Host failure rate	T_H1f_, T_H2f_	1 year
*μ*_vmmr_	VMM restart rate from downstate	T_VMM1restart_, T_VMM2restart_	1 min
*λ*_vmmf_	VMM nonaging failure rate	T_VMM1f_, T_VMM2f_	2654 hours
*δ*_vmmr_	VMM repair rate	T_VMM1repair_, T_VMM2repair_	100 mins
*β*_vmmfp_	VMM aging rate	T_VMM1fp_, T_VMM2fp_	1 month
*λ*_vmmaf_	VMM aging failure rate	T_VMM1af_, T_VMM2af_	1 week
*μ*_vmmar_	VMM recovery rate after aging failure	T_VMM1arecovery_, T_VMM2arecovery_	65 mins
*τ*_vmm_	VMM clock interval	T_VMM1clockinterval_, T_VMM1clockinterval_	1 week
*β*_vmmrej_	VMM rejuvenation rate	T_VMM1rej_, T_VMM1rej_	2 mins
*λ*_sf_	SAN failure rate	T_SANf_	1 year
*μ*_*sr*⁡_	SAN repair rate	T_SANrepair_	3 days
*λ*_vmf_	VM nonaging failure rate	T_VM1f_, T_VM2f_	2893 hours
*δ*_vmr_	VM repair rate	T_VM1repair_, T_VM2repair_	30 mins
*μ*_vmr_	VM restart rate	T_VM1restart_, T_VM2restart_	30 s
*β*_vmfp_	VM aging rate	T_VM1fp_, T_VM2fp_	1 week
*λ*_vmaf_	VM aging failure rate	T_VM1af_, T_VM2af_	3 days
*μ*_vmar_	VM recovery rate after aging failure	T_VM1arecoveryt_, T_VM2arecovery_	35 mins
*τ*_vmi_	VM clock interval	T_VM1clockinterval_, T_VM2clockinterval_	1 day
*β*_vmrej_	VM rejuvenation rate	T_VM1rej_, T_VM2rej_	1 min
*η*_vmb_	VM booting rate after VMM rejuvenation	T_VM1boot_, T_VM2boot_	30 s
*c*_ VMM_	Number of stages in *c*_VMM_-stage Erlang distribution	x	2
*c*_ VM_	Number of stages in *c*_VM_-stage Erlang distribution	x	2
*n*_ VM_	Number of VMs running on a VMM	x	2

**Table 4:** Description of case studies in steady state availability analysis.

Cases	Description
I	Rejuvenation is applied on all VMM and VM subsystems in both hosts.
II	Rejuvenation is not applied only on one of VMM subsystems in two hosts, but also on both VM subsystems in two hosts.
III	Rejuvenation is applied on both VMM subsystems in two hosts but not applied on only one of two VM subsystems.
IV	Rejuvenation is not applied on haft side of the system including VMM1 and VM1 subsystems but applied on VMM2
	and VM2 subsystems.
V	Rejuvenation is not applied on both VMM subsystems in two hosts but applied on both VM subsystems.
VI	Rejuvenation is applied on both VMM subsystems in two hosts but not applied on both VM subsystems.
VII	Rejuvenation is not applied on VMM and VM subsystems in both hosts.

**Table 5:** SSAs of VSS under given parameter values in seven case studies.

Subsystem	I	II	III	IV	V	VI	VII
VM	0.991769547666	0.991766082049	0.991770317258	0.991766912872	0.991763344539	0.991771080172	0.99176419998
VMM	0.999912470996	0.999908948744	0.999912470996	0.999908948744	0.999905284754	0.999912470996	0.999905284754

**Table 6:** VMs subsystem downtime.

Output measures	Value (hours)
Total downtime per year	72.2205464
MTTFeq	218.379208

**Table 7:** Expected number of transaction loss per year of VMs subsystem.

Main causes	Case (i): with VMM and		Case (ii): without		Case (iii): without	
	VM rejuvenation		VM rejuvenation		VMM rejuvenation	
VM nonaging failure	5.8	0.35%	5.4	1.62%	5.8	0.38%
VM aging failure	33.8	2.05%	92.2	27.66%	35.7	2.36%
VM rejuvenation	1373.9	83.28%	0	0.00%	1415.9	93.53%
VMM downtime	38.9	2.36%	38.9	11.67%	56.4	3.73%
VMM rejuvenation	196.9	11.94%	196.7	59.02%	0	0.00%

Total	1649.7	100.00%	333.3	100.00%	1513.9	100.00%
